# Single-Crystal Inspection Using an Adapted Total Focusing Method

**DOI:** 10.3390/s25103157

**Published:** 2025-05-17

**Authors:** Iratxe Aizpurua-Maestre, Aitor De Miguel, Jose Luis Lanzagorta, Ewen Carcreff, Lander Galdos

**Affiliations:** 1 IDEKO, 20870 Elgoibar, Spain; 2 TPAC, 13 Rue de la Garde, 44300 Nantes, France; 3 Faculty of Engineering, Mondragon Unibertsitatea, 20500 Mondragon, Spain; lgaldos@mondragon.edu

**Keywords:** single crystal, ultrasonic imaging, total focusing method, anisotropic materials

## Abstract

Single-crystal superalloys have attracted considerable interest in aero engine blade manufacture due to their superior mechanical properties, which maintain structural integrity at high temperatures. However, their anisotropic microstructure results in direction-dependent properties that pose a challenge for defect detection. This study proposes a methodology to determine the crystal orientation, which is subsequently used to improve the Total Focusing Method (TFM) by incorporating the refracted beam directivity. Firstly, simulations were performed using semi-analytical models (CIVA software 2023 SP4.1) to reproduce different grain orientations. The results were then post-processed to determine the grain orientation. Finally, the TFM was adapted to take into account not only the velocity variations due to orientation but also the directivity of the ultrasonic beam based only on slowness curves. The implementation of this methodology has improved the defect detection capability, optimizing the defect positioning by up to 61% and increasing the signal-to-noise ratio by up to 5 dB. This study demonstrates the effectiveness of an adapted inspection procedure for single crystals.

## 1. Introduction

In materials science, a single crystal is a material whose crystal lattice is continuous and uninterrupted throughout its volume, extending to its edges without grain boundaries, which present directionality, generating anisotropic properties [1]. In recent years, single crystals have gained considerable interest, particularly in the development of nickel-based superalloys [2]. These superalloys can operate at temperatures close to their melting point while maintaining structural integrity, making them ideal for the manufacture of turbine blades [3,4]. The NDT inspection of turbine blades is a complex and essential task that has been extensively studied by researchers using a range of NDT techniques [5]. Surface defects are typically identified by visual–optical inspection [6] and fluorescent dye penetrant methods [7], while volumetric defects are evaluated by X-ray analysis [8].

The ultrasonic method is gaining considerable attention for these types of materials [9,10]. The detection of internal defects in single crystals by ultrasonic testing is a significant challenge, because the velocity and energy distribution depend on material anisotropy [11].

**Figure 1:**
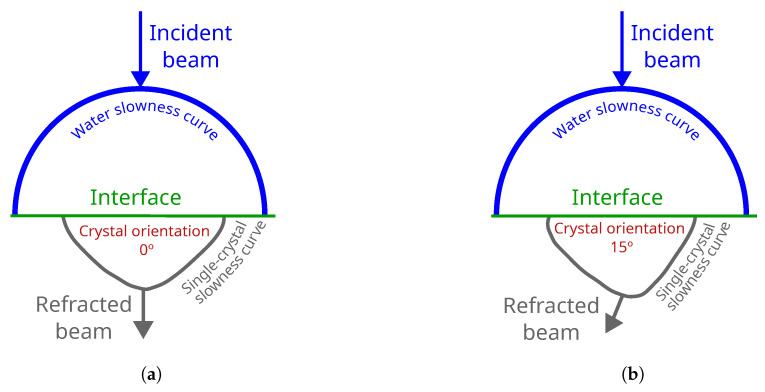
Velocity distribution in water (blue) and in the single crystal (gray) are separated by the interface (green). Considering an incidence angle of 0º (blue arrow), the refracted angle generated in the single-crystals is represented (gray arrow): (**a**) Single-crystal orientation 0°. (**b**) Single-crystal orientation 15°.

Concerning velocity distribution, the intrinsic elastic anisotropy of single crystals results in variation in the ultrasonic wave velocity with respect to the direction of propagation [12]; furthermore, this distribution characterization depends on the crystal orientation [13]. In this context, accurate determination of crystal orientation is critical. Advanced material characterization techniques [10] such as scanning electron microscopy (SEM), electron backscatter diffraction (EBSD), and transmission electron microscopy (TEM) are commonly used for this purpose. While these techniques offer high spatial resolution, their primary drawback is the labor-intensive sample preparation required [14]. Moreover, the destructive method only offers the opportunity to inspect a reduced selection of samples, while non-destructive inspections provide the possibility to inspect 100% of the components [15]. To overcome these limitations, ultrasonic inspection techniques have been developed as an alternative where the velocity variation depending on the orientation is used to determine the crystal orientation. Single-crystal orientations with ultrasound approximations have been determined on specific points with conventional ultrasonic transducers [10]. For this purpose, the velocity distribution is typically presented with slowness curves, which correlate the inverse of wave velocity (1/velocity) with the propagation direction [16]. The slowness curves are generated from a stiffness matrix using Christoffel’s equation [17]. Figure 1 summarizes the phenomena under study, where half of the slowness curve of water is represented above the interface and half of the single crystal is represented below the interface on polar axes. As shown in Figure 1a,b, the slowness curve for water exhibits a circular shape, characteristic of isotropic materials, where the wave velocity is uniform in all propagation directions. In contrast, the slowness curve for a single crystal displays a rhomboidal-like anisotropy, indicating that the wave velocity varies with propagation direction. Furthermore, as is shown in the shape of the slowness curves of the single crystals, it changes depending on the crystal orientation.

Concerning the ultrasonic beam energy distribution, the anisotropy of the component deviates the ultrasonic beam; consequently, the deviation also varies depending on the crystal orientation. Intensive work has been developed in defining the beam deviation in austenite welds with coarse structures [13,18]. These developments show the importance of considering ultrasonic beam deviation to improve defect detection and localization. The deviation of the beam can be deduced from the slowness curves generated by Christoffel’s equation [19]. In Figure 1, the refracted angle variation with the same incident beam in two different crystal orientations is represented.

The complexity of the inspection has promoted the use of advanced ultrasonic imaging techniques, allowing extensive data acquisition and the adjustment of inspection parameters according to material-specific properties [20,21]. Among these techniques, Full Matrix Capture (FMC) combined with the Total Focusing Method (TFM) [22] has previously been used. A notable application of FMC in turbine blade inspection is the use of a 2D array to detect root cracks [11,23] by Lane et al., where the anisotropic properties of CMSX-4 are taken into account, crystal orientation is quantified using EBSD, and TFM is applied, integrating the directional dependence of wave velocity and beam directivity obtained from simulations to enhance defect localization. While this approach represents a significant step forward in defect detection, current methods are still time-consuming and require extensive sample preparation.

In contrast, this study introduces a innovative ultrasonic inspection method that relies exclusively on FMC-TFM with a linear phased array (PA) transducer to evaluate single-crystal materials, specifically addressing crystalline angulation confined to the XZ plane by rotating around the Y-axis. Unlike conventional approaches, this technique requires no auxiliary characterization tools, such as EBSD or simulations, yet still delivers high-precision defect detection and localization. By extracting all necessary directional and structural information from ultrasonic data, this streamlined method marks a significant leap forward in inspecting complex anisotropic materials with greater efficiency, accuracy, and simplicity. First, it establishes a novel method for determining grain orientation based on the amplitude and Time-of-Flight (TOF) distribution of the backwall echo. By performing pulse–echo inspections at discrete points, variations in amplitude and TOF related to the crystal’s angular orientation can be detected [10]. Nevertheless, discrete point measurements may be influenced by factors such as variations in water path and attenuation due to surface roughness, which can distort the ultrasonic response [24]. To enhance inspection reliability, this study replaces discrete inspection points with a novel approach considering multiple emitter–receiver combinations from the FMC technique. By leveraging these combinations, the beam width (W) is characterized for each crystal orientation. The second contribution focuses on adapting the TFM method to the identified crystal orientation, incorporating beam energy directivity based on the slowness curve. While previous studies have relied on beam simulations to determine directivity [20], the present approach eliminates the need for such simulations. Instead, the adaptation is directly defined by the properties of slowness curves. In these optimized reconstructions, only the stiffness matrix of the single crystal and the crystal orientation (determined in the first contribution) are required. This development presents the complete process to perform the inspection of single crystals based on ultrasound results.

## 2. Methodology

**Figure 2:**
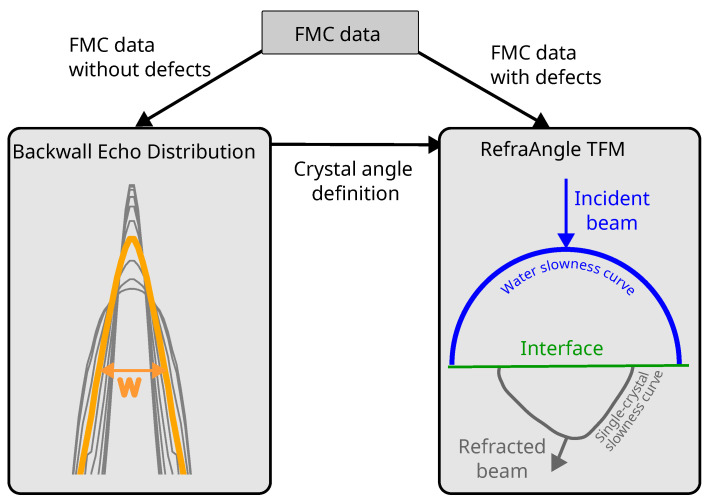
Inspection procedure definition. FMC data without defects is generated to characterize the width of the backwall echo distribution depending on the crystal orientation angle to identify the re orientation. FMC data with defects corresponding to each crystal orientation has been generated to determine the inspection capability of adapted TFM algorithms for each orientation.

To assess the ultrasonic inspection of single-crystal materials, simulation data were generated using the FMC technique in CIVA software (see Figure 1). The inspection was performed both in the presence and absence of defects. The FMC data obtained from defect-free simulations were utilized to analyze the backwall echo amplitude and TOF distribution, allowing the characterization of the beam width (W) as a function of the single-crystal orientation. Subsequently, the TFM reconstruction was adapted using FMC data from simulations with defects, incorporating the slowness curves corresponding to each crystal orientation (see Figure 2).

### 2.1. FMC Data Generation

**Figure 3:**
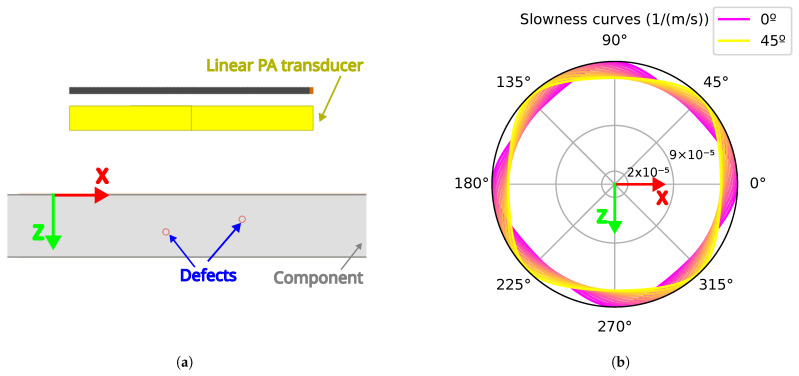
Simulation definition: (**a**) Set up where the ultrasound inspection is performed with a linear phased array in immersion to detect the two defects defined in the component. (**b**) The variation in slowness curves is illustrated using crystal orientations ranging from 0º (purple) to 45º (yellow), in 5º increments. The color transition from purple to yellow visually represents the increasing orientation angle.

The inspection data were obtained by simulation using a linear PA transducer in immersion, both with and without the two defects represented at different depths (see Figure 3a). A material with cubic symmetry was modeled by specifying the three independent coefficients of the stiffness matrix and varying the crystal orientation from 0° to 45° in 5° increments within the X-Z plane. The corresponding slowness curves are shown in Figure 3b. For more details, see Table 1.

### 2.2. Backwall Echo Distribution

As mentioned above, the defect-free simulations were used to define the backwall echo distribution. The following procedure was followed:

- From the ultrasonic data matrix generated by the FMC technique, the results obtained for each crystal angulation were compared by analyzing the Ascans corresponding to *tx = N − rx* combinations, where *tx* refers to the transmitting element, *rx* to the receiving element and *N* to the number of elements of the linear PA.
- In each Ascan, the response of the backwall echo was extracted by defining a gate from $t_{1}$ to $t_{2}$ based on the thickness of the component.
- In the Ascan of each combination, the maximum of the gate was determined, in amplitude (see Equation (1)) and TOF (see Equation (2)) based on the emitter element, where *A(tx)* corresponds to the maximum backwall amplitude and *TOF(tx)* to the position of the maximum amplitude.
  (1) $$A\left(tx\right)=\sum_{tx=1}^{N}\max\left(Ascan_{tx}\left(t_{2}-t_{1}\right)\right),\;\mathrm{where}\;tx=N-rx$$
  (2) $$TOF\left(tx\right)=\sum_{tx=1}^{N}argmax\left(Ascan_{tx}\left(t_{2}-t_{1}\right)\right),\;\mathrm{where}\;tx=N-rx$$
- The different widths of the generated ultrasonic beam were determined based on −6 dB attenuation of the maximum signal. The transmitters that produced a response with a 6 dB attenuation from the maximum were identified based on Equation (3).
  (3) $$T_{-6\mathrm{dB}}=\left\{tx\in T\;|\;A\left(tx\right)\geq \max\left(A\right)-6\;\mathrm{dB}\right\}$$
  After selecting the transmitter elements, the width was obtained by Equation (4), where *p* refers to pitch dimension (0.6 mm, as indicated in Table 1).
  (4) $$W=(tx_{2}-tx_{1})\cdot p$$

### 2.3. TFM Algorithm Implementation

**Figure 4:**
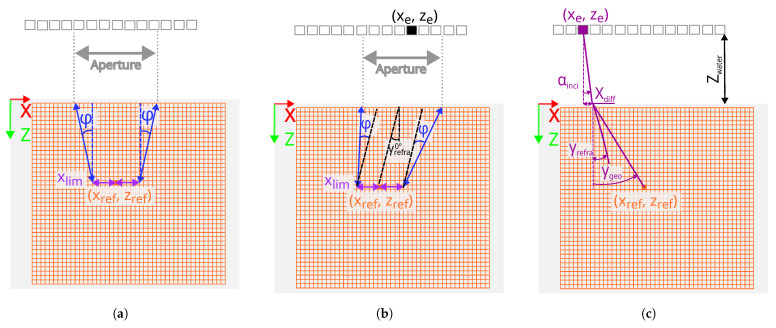
Filter definition: (**a**) F-number. (**b**) RefraFilter. (**c**) RefraCons.

The classical approaches to TFM reconstruction, namely “Strategy Iso” and “Strategy Ani”, were first computed to establish the baseline for the inspection framework. Subsequently, two distinct strategies, A and B, were developed to enhance these classical approaches. “Strategy A” introduces an advanced filter, RefraFilter, based on the refraction angle, while “Strategy B” presents a novel methodology, RefraRecons, for determining the water/solid interface point, replacing conventional approaches. Table 2 represents the hypotheses considered in each approximation, which focus on the following three features:

- Velocity: The stiffness matrix defined in Table 1 was used to calculate the slowness curves by Christoffel’s Equation (5), where, $\delta_{ik}$ is the Kronecker symbol, *V* is the phase velocity, *n* is the propagation direction, and $C_{ijkl}$ is the single-crystal stiffness matrix.
  (5) $$\rho V^{2}\delta_{iK}-C_{ijkl}n_{j}n_{k}=0$$
  On the one hand, the isotropic approach assumes a constant mean velocity of slowness curves. On the other hand, the anisotropic approach considers velocity variations as a function of the propagation angle.
- Filter: The filter defines the number of elements or aperture considered in each reconstruction grid. In the classical approaches, the conventional filters $X_{\lim}$ and slope (*tan (φ)*) [27] are considered. $X_{\lim}$ and slope are defined based on the beam divergence. In Figure 4a, the linear transducer and the component with the reconstruction grid are presented. The grid ($x_{\mathrm{ref}}$, $z_{\mathrm{ref}}$) has been highlighted and the aperture has been calculated based on $X_{\lim}$ and slope parameters. As shown in Figure 4a, the filter is symmetrical to the grid point. On the other hand, in the proposed approaches,“Strategy A” and “Strategy B”, the RefraFilter (see Figure 4b) has been defined, which is not symmetrical with respect to the pixel. The contribution of the RefraFilter to the optimization of the TFM reconstruction is explained in detail in “Strategy A”.
- Interface: For the classical approaches and “Strategy A”, the interface point of the beam between the coupling medium and the component was calculated using Fermat’s minimum time principle. According to this principle, the wave propagation through the water/solid interface must correspond to the minimum propagation time [28,29]. To further optimize inspection results, “Strategy B” introduces RefraRecons, which is the second key contribution aimed at enhancing the effectiveness of the inspection process. The contribution of RefraRecons is explained in detail in “Strategy B”.

Once the general framework of the methodology based on the three key elements (velocity, filter, and interface) has been established, specific details of the new approaches are described in depth below:

- Strategy A: The Fermat principle of minimum time and velocity variation have been calculated as in “Strategy Ani”. However, in order to reduce the noise considering the beam directivity, the RefraFilter has been designed based on the refraction angle at a 0° incidence angle instead of using the conventional filters $X_{\lim}$ and slope (*tan* (φ)). The refraction angle at 0° depends on the single-crystal orientation and it has been calculated based on Christoffel’s equation and the Fermat principle of stationary time considering the phase velocity. The Fermat principle of stationary time states that at the interface between two media, the horizontal component of phase slowness must remain continuous across the interface. This property must be preserved for both isotropic and anisotropic media regardless of the nature of the waves generated in the boundary [28,30]. Figure 4b represents a linear PA transducer with a component with a grid where a point ($x_{\mathrm{ref}}$, $z_{\mathrm{ref}}$) has been highlighted. In addition, $\gamma_{\mathrm{refra}}$ at a 0° angle of incidence is represented and the filter is defined based on this. The filters described in Equations (6) and (7) have been applied, where $x_{\mathrm{ref}}$ and $z_{\mathrm{ref}}$ correspond to each position of the TFM reconstruction grid, $x_{e}$ corresponds to the PA elements at the X position, and $\gamma_{\mathrm{refra}}^{0^{\circ }}$ corresponds to the refracted angle at 0°.
  (6) $$x_{\mathrm{ref}}-x_{e}\geq -X_{\lim}-\tan\left(\varphi -\gamma_{\mathrm{refra}}^{0^{\circ }}\right)\cdot z_{\mathrm{ref}}$$
  (7) $$x_{\mathrm{ref}}-x_{e}\leq X_{\lim}+\tan\left(\varphi +\gamma_{\mathrm{refra}}^{0^{\circ }}\right)\cdot z_{\mathrm{ref}}$$
- Strategy B: The variation in the refraction angle as a function of the crystal orientation has been taken into account, as in “Strategy Ani” and “Strategy A”. However, a different method has been implemented to determine the water/solid interface point, replacing the use of Fermat’s minimum time principle. In “Strategy B”, the interface point is identified as the location that produces a refracted angle closest to the energy refracted angle. Iteration with different incident angles has been performed to determine the interface point between the water and the component. To achieve this, the incidence angle has been varied from −5° to 5° in 0.05° increments. Equation (8) defines the $X_{\mathrm{diff}}$, which is the X-axis distance of the beam in the water, where $\alpha_{\mathrm{inci}}$ corresponds to the incidence angle in the water and $Z_{\mathrm{water}}$ corresponds to the water distance in the *Z*-axis (see Figure 4c).
  (8) $$X_{\mathrm{diff}}\left(\alpha_{\mathrm{inci}}\right)=\tan\left(\alpha_{\mathrm{inci}}\right)\cdot Z_{\mathrm{water}},\;\alpha_{\mathrm{inci}}\in \left[-5^{\circ },5^{\circ }\right],\;\mathrm{with}\;a\;\mathrm{step}\;\mathrm{of}\;0.05^{\circ }.$$
  For each incidence angle, a geometrical angle in the component, $\gamma_{\mathrm{geo}}$, has been defined (see Equation (9)), where $x_{\mathrm{ref}}$ and $z_{\mathrm{ref}}$ correspond to the X and Z positions of the reconstruction pixel, and $x_{e}$ corresponds to the PA elements’ X position (see Figure 4c).
  (9) $$\gamma_{\mathrm{geo}}\left(\alpha_{\mathrm{inci}}\right)=\arctan\left(\frac{x_{\mathrm{ref}}-x_{e}-X_{\mathrm{diff}}\left(\alpha_{\mathrm{inci}}\right)}{z_{\mathrm{ref}}}\right)$$
  The geometrical angle,$\gamma_{\mathrm{geo}}$, has been compared to the refracted angle, $\gamma_{\mathrm{refra}}$, obtained through slowness curves applying the Fermat principle of stationary time for each incidence angle. The incidence angle, which presents the minimum difference, has been selected, $\alpha_{\mathrm{inci}}^{\mathrm{opt}}$ (see Equation (10)). The ultrasonic path has been calculated with the selected incident angle.
  (10) $$\alpha_{\mathrm{inci}}^{\mathrm{opt}}=\arg\min_{\alpha_{\mathrm{inci}}}\left|\gamma_{\mathrm{geo}}\left(\alpha_{\mathrm{inci}}\right)-\gamma_{\mathrm{refra}}\left(\alpha_{\mathrm{inci}}\right)\right|$$
  Concerning the filters, the same filter applied in “Strategy A” has been introduced (see Equations (6) and (7).

## 3. Results and Discussion

### 3.1. Crystal Angle Definition: Backwall Beam Distribution

**Figure 5:**
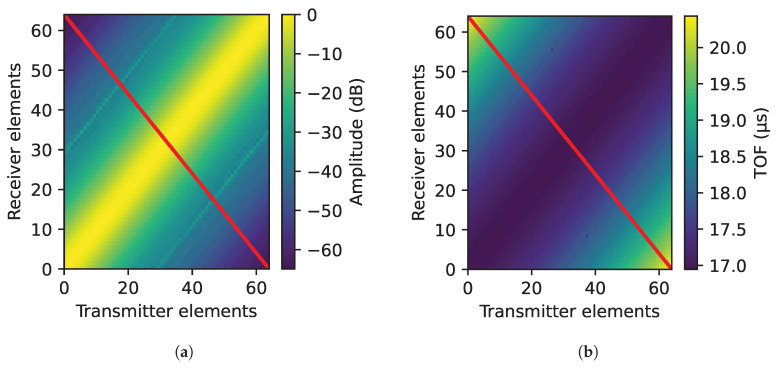

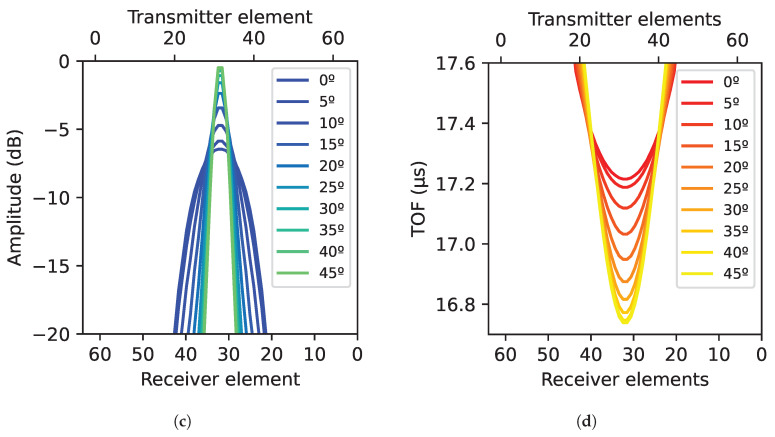
Backwall echo distribution: (**a**) Backwall amplitude distribution for all emitter–receiver combinations for a crystal angle of 20°. (**b**) Backwall TOF distribution for all emitter–receiver combinations for a crystal angle of 20°. (**c**) Backwall amplitude distribution of the combinations selected in Equation (1) for the analyzed crystal orientations. (**d**) Backwall TOF distribution of the combinations selected in Equation (1) for the analyzed crystal orientations.

As an example, the backwall echo amplitude and TOF distribution for all emitter–receiver combinations at a crystal orientation of 20° are presented (see Figure 5a,b). The Y-axis represents receiver elements while the X-axis represents the emitter elements. For the amplitude distribution (see Figure 5a), it peaks along the diagonal, where the emitter and receiver are closed to each other, and gradually decreases with the distance between the emitter and receiver. Conversely, the TOF (see Figure 5b) reaches a minimum along the diagonal, where the emitter and receiver are close, and increases beyond it. With the objective to compare the results to each other, the emitter–receiver combinations of the red line have been extracted and are shown in Figure 5c,d, where the backwall echo has a characteristic distribution depending on the orientation of the single crystal in amplitude (see Equation (1)) and TOF (see Equation (2)), respectively.

**Figure 6:**
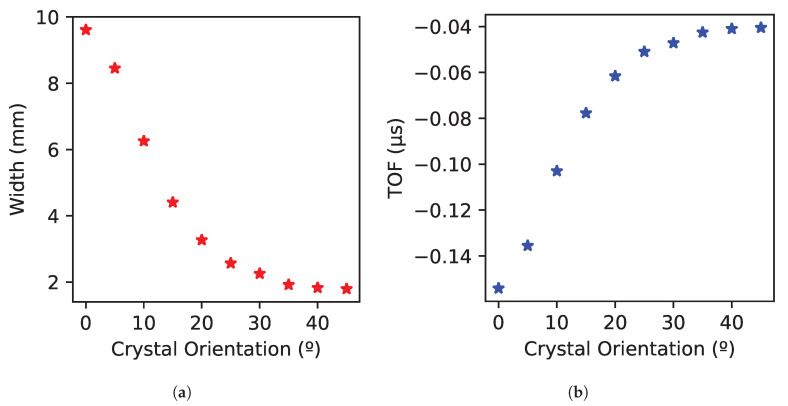
(**a**) Width calculation. The stars represent the width obtained with Equation (4) for each crystal orientation (**b**) TOF calculation. The stars represent the difference in TOF from the answer of the element defined in Equation (3) to the maximum answer in amplitude for each crystal orientation.

The width definition based on 6 dB attenuation (see Equation (4) is represented in Figure 6a. In addition, TOF variation of the signals representing the amplitude attenuation of −6 dB is shown in Figure 6b.

**Figure 7:**
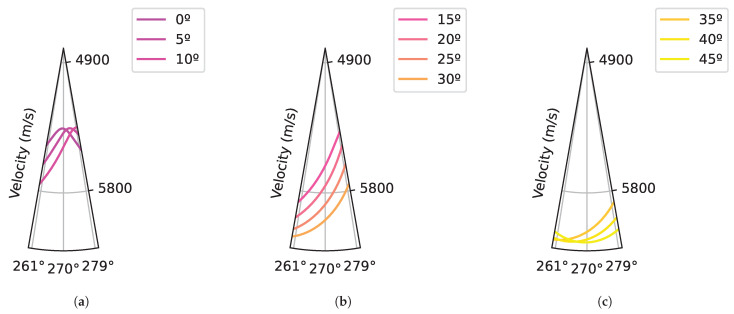
Velocity distribution depending on the crystal orientation (**a**) Low angles. (**b**) Middle angles. (**c**) High angles.

Based on Figure 6a, the width changes from 9.8 mm to 1.7 mm depending on the orientation, and it is more noticeable at orientations with low angles than at high angles. Furthermore, the distribution variation is really small at angles ranging from 35° to 45°. Concerning the difference in TOF, the variation (see Figure 6b) is also low, from 0.05 μs to 0.15 μs, especially at high angles. This is because the velocity changes less at angles from 35° to 45° than at low angles (see Figure 7). Therefore, experimental testing has been considered necessary to ensure implementation at high angles between 35° and 45°.

### 3.2. TFM Algorithm Reconstruction

**Figure 8:**
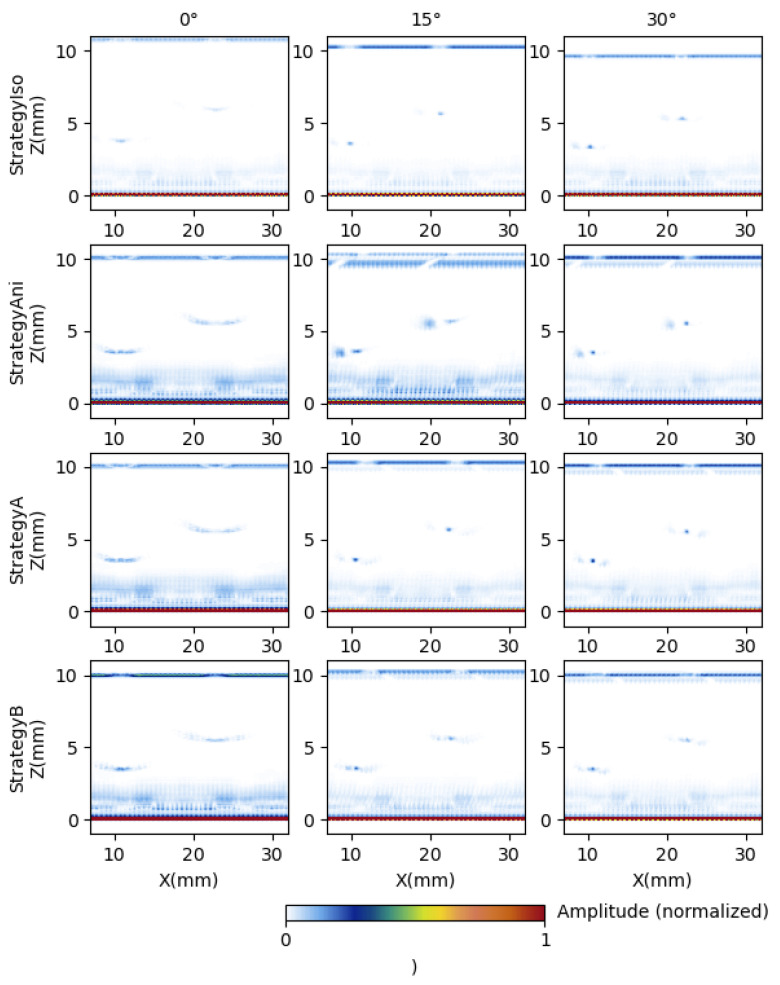
Results of single-crystal angles of 0°, 15°, and 30° with TFM reconstruction strategies “Strategy Iso", “Strategy Ani”, “Strategy A”, and “Strategy B”. The colors represent the amplitude of the answers.

The classical approaches “Strategy Iso” and “Strategy Ani” as well as the proposed “Strategy A” and “Strategy B” are represented in Figure 8 for three different crystal orientations (0°, 15°, and 30°). In each of the representations, the surface echo is located at Z = 0 mm and the backwall echo around Z = 10 mm. In addition, the defect response is visualized in all the reconstructions around Z = 4 mm (Defect 1) and Z = 6 mm (Defect 2). Concerning the crystal orientation of 0°, it corresponds to the direction with the smallest velocity; therefore, the backwall echo is not visible in “Strategy ISO”. Focussing on “Strategy Ani” and “Strategy A”, the refraction angle at 0° is nearly 0°; consequently, the answers are similar. The results have improved in the case of “Strategy B”. Concerning the crystal orientations of 15° and 30°, they have a refraction angle of 16.5° and 13.5° at 0°, respectively. Consequently, an improvement is noticed in “Strategy A” compared to “Strategy Any”. Focusing on “Strategy B”, the noise distribution changes compared to “Strategy A”.

**Figure 9:**
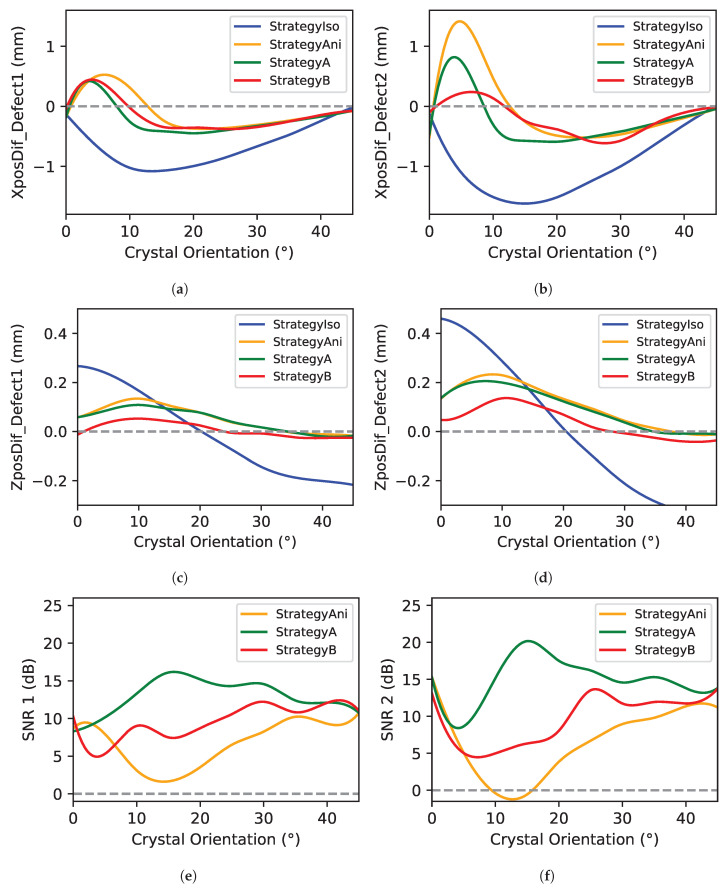
The reconstruction value analysis: (**a**) Deviation of the X position in defect 1. (**b**) Deviation of the X position in defect 2. (**c**) Deviation of the Z position in defect 1. (**d**) Deviation of the Z position in defect 2. (**e**) SNR in defect 1. (**f**) SNR in defect 2.

Based on the previous TFM reconstruction, the positioning accuracy and signal-to-noise ratio (SNR) of each defect have been quantified (see Figure 9). It is important to note that the SNR considers the maximum defect response and noise within a region not exceeding 3 mm. In Equation (11), the measurement of the SNR is represented, where $A_{\mathrm{defect}}^{\max}$ represents the maximum amplitude of the defect and $A_{\mathrm{noise}}^{\max}$ represents the highest amplitude of the noise exceeding 3 mm around the defect.

(11) $$\mathrm{SNR}=20\log_{10}\left(\frac{A_{\mathrm{defect}}^{\max}}{A_{\mathrm{noise}}^{\max}}\right)$$

Regarding the variation in defect 1 and defect 2 in X positioning (see Figure 9a,b), in defect 1, the strategies that consider the anisotropic velocity variation of the ultrasonic beam (“Strategy Ani”, “Strategy A”, and “Strategy B”) provide similar answers compared to “Strategy Iso”, which considers the mean velocity as the unique propagation velocity. It is evident that the use of the mean velocity results in a defect response with poor positional accuracy, exceeding 1 mm. In the case of defect 2, the deviation of “Strategy Ani” is higher than the proposed “Strategy A” and “Strategy B” at low angles (close to 5°).

Considering the deviation in the Z position of defect 1 and defect 2 (see Figure 9c,d), as in the case of the X positioning deviation, "Strategy Iso” gives the worst answers by far. Among the rest of the strategies, “Strategy Ani” and “Strategy A” provide similar answers, because they are considering the same ultrasonic beam path calculated with the Fermat principle of minimum time. However, “Strategy B” yields excellent results with minimal deviation from the ideal Z position. The application of the minimum refracted angle difference, rather than using Fermat’s principle, leads to superior Z positioning, as it accounts for the energy propagation path. To quantify the improvement, it is important to note that the highest deviation in “Strategy Ani” is 0.13 mm for defect 1 and 0.23 mm for defect 2, whereas the highest deviation in “Strategy B” is 0.05 mm for defect 1 and 0.13 mm for defect 2. The answer of “Strategy B” is worse in angles between 30°and 45°. It is assumed that this is a consequence of small velocity changes depending on the angle (see Figure 7), which generate small refraction angles, which do not have enough influence on the energy distribution. However, this requires further analysis.

Finally, concerning the SNR of defects 1 and 2 (see Figure 9e,f), it is important to note that the isotropic values were not considered in the analysis due to the poor results obtained in defect location. In general, the proposed “Strategy A” and “Strategy B” present a higher SNR than “Strategy Ani”. Among the two proposed strategies, “Strategy A” exhibits a higher SNR than “Strategy B,” particularly in response to defect 2, where the SNR improvement is considerable. It is interesting to note that the highest SNR of “Strategy Ani” is 15 dB, whereas the maximum SNR in “Strategy A” is 20 dB. This happens because “Strategy Ani” does not implement any kind of filter considering the energy path; consequently, the reconstruction suffers from distortion (see Figure 8, “Strategy Ani” and crystal angle “15°”) and noisy areas close to the defects (see Figure 8, “Strategy Ani” and crystal angle “15°”).

The proposed methodology to perform the single-crystal inspection based on the FMC-TFM technique demonstrated its feasibility. Concerning the crystal orientation definition, instead of considering single answers [10,31], the use of different emitter–receiver combinations provides the possibility to acquire more data to increase the reliability of the inspection. Concerning the TFM algorithm adaptation based on refraction angles, it shows an improvement of 61% in Z positioning and an improvement of 5 dB in the SNR, without the need to perform simulations for beam directivity definition [11].

In this development, a specific material with a cubic anisotropy has been analyzed in the XZ plane; however, the presented procedure can be extrapolated to other materials that are homogeneously anisotropic, such as composite materials [32] or forged materials [33]. Additionally, the improvements of the presented procedure are more important in crystal orientation, which distorts the beam direction more notoriously, for example, in a crystal orientation of 15°. Consequently, it is deduced that applying the presented procedure in highly anisotropic materials can be suitable. Nevertheless, it requires a deeper analysis. Regarding the procedure applicability in complex geometries, in case of inspection surfaces with fixed curvature, this procedure can be applied, including the variability in incidence angle. In case of more complex or unknown surfaces, a feasibility study to discern the the geometry influence from the crystal orientation is required for crystal angle definition [34]. Although experimental tests are necessary to validate the technique, in light of the results, the application of this new methodology could serve as the foundation for developing new FMC-TFM algorithms to accurately detect defects in materials with anisotropic behavior.

## 4. Conclusions

Overall, this study proposes a procedure for single-crystal inspection based on FMC data analysis for crystal angle orientation and TFM reconstruction, considering the refracted angle to improve volumetric defect detectability. The procedure requires only inspection data from different crystal angles and the stiffness matrix of the component.

Using FMC data analysis, the development has demonstrated that the amplitude and TOF distribution of the backwall echo considering specific emitter–receiver combinations of the FMC technique can be used to determine the single-crystal orientation with a variation in width from 9.8 mm to 1.7 mm and a variation in TOF from 0.15 μs to 0.05 μs.

Considering the TFM reconstruction, the development has demonstrated that the proposed “Strategy A” and “Strategy B”, which consider the refracted angle calculated from slowness curves, optimized the answer compared to “Strategy Ani”, which is not adapted based on the refracted angle. “Strategy A”, which modifies the emitter–receiver combinations interfering at each point of the grid based on the refracted angle at 0°, has increased the SNR by 5 dB compared to “Strategy Ani”. Furthermore, the positioning has also been increased. “Strategy B”, which defines the interface point between water and the component by minimizing the angular difference between the propagation angle and expected refracted angle in the component, has optimized Z-axis deviation by 61% and has increased the SNR in defect 2 compared to “Strategy Ani”.

It has been shown that this process provides guarantees for its implementation, reducing the number of inspection methods implemented in a single-crystal inspection and providing more accurate results.

## Figures and Tables

**Table 1:** Simulation parameter definition.

Component	Linear PA Transducer	Defects
Thickness: 10 mm	Pitch: 0.6 mm	Diameter: 1 mm
C11/C12/C44:	Frequency: 25 MHz	Depths 4 and 6 mm
250/160/124 Gpa ^1^	Elevation: 10 mm	
Density: 8.72 g/cm^3^ ^2^	Number of elements: 64	

^1^ The anisotropy of CMSX-4 material has been obtained from references [25,26]. ^2^ The density of CMSX-4 material has been obtained from reference [23].

**Table 2:** Summary of the hypothesis of the TFM reconstruction approaches. The lines corresponds to analyzed hypothesis and the columns to implemented strategies. The hypothesis implemented in each strategy are identified with an "X".

		Classical Approaches		Proposed Approaches	
		Strategy Iso	Strategy Ani	Strategy A	Strategy B
Velocity	Isotropic	X			
	Anisotropic		X	X	X
Filter	F-number	X	X		
	RefraFilter			X	X
Interface	Fermat Princ.	X	X	X	
	RefraRecons.				X

## Data Availability

The original contributions presented in this study are included in the article material. Further inquiries can be directed to the corresponding author.
